# A Novel Promising Frontier for Human Health: The Beneficial Effects of Nutraceuticals in Cardiovascular Diseases

**DOI:** 10.3390/ijms21228706

**Published:** 2020-11-18

**Authors:** Albino Carrizzo, Carmine Izzo, Maurizio Forte, Eduardo Sommella, Paola Di Pietro, Eleonora Venturini, Michele Ciccarelli, Gennaro Galasso, Speranza Rubattu, Petro Campiglia, Sebastiano Sciarretta, Giacomo Frati, Carmine Vecchione

**Affiliations:** 1 Department of Angio-Cardio-Neurology, IRCCS Neuromed, 86077 Pozzilli, Italy; albino.carrizzo@gmail.com (A.C.); maur.forte@gmail.com (M.F.); eleonora.venturini94@libero.it (E.V.); speranzadonatella.rubattu@uniroma1.it (S.R.); sebastiano.sciarretta@uniroma1.it (S.S.); fraticello@inwind.it (G.F.); 2 Department of Medicine and Surgery, University of Salerno, 84081 Baronissi, Italy; carmine.izzo93@gmail.com (C.I.); pdipietro@unisa.it (P.D.P.); mciccarelli@unisa.it (M.C.); ggalasso@unisa.it (G.G.); pcampiglia@unisa.it (P.C.); 3 Department of Pharmacy, University of Salerno, 84084 Fisciano, Italy; esommella@unisa.it; 4 Department of Clinical and Molecular Medicine, School of Medicine and Psychology, Sapienza University of Rome, Ospedale S.Andrea, Via di Grottarossa 1035, 00189 Rome, Italy; 5 Department of Medico-Surgical Sciences and Biotechnologies, Sapienza University of Rome, Corso della Repubblica 74, 04100 Latina, Italy

**Keywords:** resveratrol, cocoa, quercetin, curcumin, brassica, berberine, Spirulina platensis, CVDs

## Abstract

Cardiovascular diseases (CVDs) such as hypertension, atherosclerosis, myocardial infarction, and diabetes are a significant public health problem worldwide. Although several novel pharmacological treatments to reduce the progression of CVDs have been discovered during the last 20 years, the better way to contain the onset of CVDs remains prevention. In this regard, nutraceuticals seem to own a great potential in maintaining human health, exerting important protective cardiovascular effects. In the last years, there has been increased focus on identifying natural compounds with cardiovascular health-promoting effects and also to characterize the molecular mechanisms involved. Although many review articles have focused on the individual natural compound impact on cardiovascular diseases, the aim of this manuscript was to examine the role of the most studied nutraceuticals, such as resveratrol, cocoa, quercetin, curcumin, brassica, berberine and Spirulina platensis, on different CVDs.

## 1. Introduction

Cardiovascular diseases (CVDs) represent the leading cause of death globally [1,2,3]. It is well known that the common cardiovascular risk factors such as advanced age, hypertension, diabetes, hypercholesterolaemia, left ventricular hypertrophy, and heart failure are the main contributors of cardiovascular complications [4,5,6,7,8,9]. Unfortunately, the current pharmacological therapies used for the management of CVDs often failed to be effective in patients who are reluctant to undergo multiple-drug regimens, so adherence to therapy is reduced. Thus, an integrative approach capable of improving cardiovascular protection is urgently needed.

In the last decade, the food industry has focused on the characterization and identification of bioactive molecules contained in foods or in natural matrices, which, besides their nutritional value, can bring health benefits, especially in the treatment of chronic diseases. This process started mainly because these natural compounds are characterized by fewer side effects in comparison with pharmacological therapies, so consumers tend to prefer their use for health promotion. Based on these considerations, the last ten years saw the rapid expansion and commercialization of a new class of products, which are based on an enriched or concentrated mixture of bioactive compounds of natural origin, with several healthy claims that are experimentally verified, these are the so-called “nutraceuticals”. These products derive from the term “Nutraceutics” coined by Dr. De Felice in 1989, and they refer to a group of products that fall in the gap between nutrition and pharmacological therapy. Nutraceuticals (from the terms “nutrition” and pharmaceuticals”) are defined as foods or part of foods that share health-promoting effects (“pharmaceutic properties”) and include polyphenols, carotenoids, polyunsaturated fatty acids, and natural peptides. Compared to therapeutics, nutraceuticals do not have patent protection and governmental sanction and may be used either as preventive agents or as adjuvants of traditional therapy. Although the definition of nutraceuticals in part overlaps that of functional foods and the two terms are often considered synonymous, functional foods may be defined as all foods fortified with compounds aimed to enhance nutritional or healthy potential and are not sold in dosage forms, whereas nutraceuticals exits also in form of tablets, capsules, or powder [10,11]. This marks also the line with respect to dietary supplements, which are usually products that aim to integrate the diet with macro or micronutrients such as amino acids, minerals, and vitamins. In this regard, several natural products represent also a source of vitamins and trace elements. However, many clinical trials failed to show preventive or therapeutic beneficial effects of vitamins in CVDs [12,13].

The importance of nutrition in regulating blood pressure is well documented [9,14,15,16,17,18]. Beyond the reduction in blood pressure values shown for the “Mediterranean diet” and the “DASH Diet”, in recent years, several studies have documented the antihypertensive action of numerous well-known phytochemicals, which are highly present in nutraceutical formulations, such as cocoa flavonoids, which have been demonstrated to reduce systolic and diastolic blood pressure by about 4–5 mmHg and 2–3 mmHg, respectively).

Taking into consideration the potential efficacy, despite the incompleteness of the scientific information available for many nutraceutical active ingredients, in the last few years, the scientific community has gathered a considerable interest in the characterization and validation of natural compounds in CVDs by investigating their molecular mechanisms and initiating numerous clinical trials.

This review will exemplify the role of different natural compounds in several CVDs, focusing on resveratrol, cocoa, quercetin, curcumin, brassica, berberine and Spirulina platensis, highlighting their mechanistic role and the data, until now, available in humans.

## 2. Nutraceuticals in Hypertension

Hypertension is one of the most important risk factor for CVDs and represents the main contributor for increased death rates in industrialized countries [19,20,21,22]. The life-long risk of developing hypertension is estimated at 90%, and a correct pre-pathologic non-pharmacologic approach can be of help to undermine all its complications. Moreover, hypertension is an insidious pathology, as its symptoms are difficult to detect. As a consequence, before correctly approaching this pathology, the hypertensive organ damage has time to develop [23,24,25,26,27,28].

To date, many drugs have been used to correct high blood pressure (BP) levels in order to reduce the incidence of cardiovascular complications and accidents. However, as guidelines show, not all types of hypertension are to be pharmacologically treated, especially in case of borderline levels (Systolic Blood Pressure 130–139 mmHg–Diastolic Blood Pressure 85–89 mmHg) [29,30,31,32].

In this view, nutraceuticals in association with other lifestyle interventions and diet seem to be the new frontier. Therefore, it becomes important to discover all those micronutrients that can help prevent hypertension, thus balancing out our everyday life [32,33].

### 2.1. Resveratrol

Resveratrol (3,4,5-trihydroxy-trans-stilbene) (RES) is a powerful antioxidant present in significant concentrations in grape skin [34]. Many studies have shown antihypertensive effects of RES in various preclinical hypertensive models through various mechanisms including, in addition to the antioxidant action, the stimulation of endothelial nitric oxide (NO) production, the inhibition of vascular inflammation and the prevention of platelets aggregation [35,36]. Interestingly, besides the reduction in BP levels, RES was also reported to be protective in animals with dyslipidemias and insulin resistance and to reduce cardiac hypertrophy and contractile dysfunction [37,38,39,40,41]. The mechanisms underlying the beneficial effects of RES are in most cases endothelium-dependent and involve the AMPK, SIRT-1, and Nrf2 pathways, all of which are associated with an improved availability of NO (by increased eNOS activity), reduction in vascular smooth cell contractility (via inhibition of Ang-II) [37,42,43,44]. Among human trials, the administration of RES in double-blind, randomized, placebo-controlled clinical trials showed a decreased expression of endothelial cell ICAM, VCAM, and IL-8 as well as of inflammatory markers [45]. Recently, RES-enriched nutraceuticals have also shown the ability to reduce triethylamine-N-oxide (TMAO) serum levels in a randomized, placebo-controlled, cross-over trial. TMAO has gained interest as a marker of CVDs. After 4 weeks, the level of TMAO was significantly decreased in the treatment group compared to placebo (63.6% vs. 0.54%) [46].

Few studies have attempted to highlight the ability of RES to significantly reduce BP levels. Some studies did not support any evidence for BP reduction, which was probably due to a low bioavailability in humans or to a lack of direct efficacy [47,48,49,50]. In contrast, a meta-analysis showed that at high doses, >150 mg/day, RES not only reduced BP levels but also improved the overall metabolic parameters [51]. Of interest, RES effectiveness increases in patients with dyslipidemias as observed in animal studies. This evidence is of great importance for the overall patient’s welfare [38,52]. A new field of interest is the association of RES with the standard antihypertensive treatment. A better BP control and a significant reduction in SBP and DBP in patients undertaking this treatment combination has been highlighted [53]. The effect of RES in combination with recommended treatment on BP in patients with prehypertension and stage 1 hypertension is currently under investigation [54].

### 2.2. Cocoa

Daily intake of flavonoids is associated with a decrease in the risk of developing coronary heart disease, stroke, and overall CVDs [55]. A high number of food flavonoids have vascular protective effects, through their antioxidant and anti-inflammatory properties, improving NO metabolism and endothelial function [56,57]. Cocoa, which is particularly rich of oligomeric procyanidins, is among the most studied nutraceuticals: in particular, chocolate flavonoids appear to increase the bioavailability of NO, protecting the vascular endothelium and decreasing the levels of some risk factors for CVDs such as insulin resistance and systemic inflammation through increased plasma antioxidant capacity [57,58]. Clinical trials showed the ability of cocoa to increase peripheral vasodilatation, improve antioxidant status, and decrease BP. An overall amelioration of coronary function following cocoa administration has been reported in humans. In this regard, the enhancement of coronary vasodilatation and the improvement of coronary endothelial function have been observed, along with an overall increased coronary vasodilatation in terms of coronary velocity flow reserve [59,60,61,62,63,64,65,66,67]. In healthy subjects, the administration of 821 mg/d flavanol-rich cocoa for 5 days showed an improvement of peripheral vasodilatation and an improvement of vasodilatation response to ischemia, as assessed by pulse-wave amplitude on the finger [59]. On the same basis, 2 weeks of high-flavonoid chocolate vs. low-flavonoid chocolate administration in healthy subjects showed an improvement of flow-mediated vasodilatation of brachial artery and a consensual increase in plasma epicatechin concentration [61]. More interestingly for future considerations, healthy subjects with at least one cardiovascular risk factor have shown an improvement in flow-mediated vasodilatation and an increase in nitrosated and nitrosylated species levels after a 15-day cross-over administration of 100 g dark chocolate with 21.91 mg catechin and 65.97mg epicatechin vs. flavanol-free white chocolate [64]. A similar improvement in flow-mediated vasodilatation has been detected in smoker and diabetic subjects under both acute and chronic administration [65,68]. Although the great heterogeneity in the characteristics of trials and the recruitment of subjects, mostly healthy, and the need to better characterize the vascular effects of Cacao, the literature data suggest the potential of cocoa as a promoter of the cardiovascular health.

### 2.3. Quercetin

Quercetin is another flavonoid that has been shown to be effective as an antihypertensive molecule. Quercetin shows several functions, such as anti-inflammatory, through interleukin and TNF-α pathways, and anti-oxidative, through cyclooxygenase/lipoxygenase pathways, and it also acts as a scavenger agent, maintaining BP levels through vascular compliance and elastic resistance. It is also able to downregulate the autonomic nervous system, which is a basal cause of hypertension [69,70,71,72,73]. Quite interesting is the ability of quercetin to regulate total blood volume by the reduction of ENaC gene expression, decreasing body fluid volume [74]. In particular, a quercetin-induced stimulation of NKCC1 regulates transepithelial Chloride-secretion, ultimately resulting in a reduction of ENaC mRNA expression. The reduced production of ENaC protein leads to a reduced ENaC-related Na+-reabsorption. ENaC (epithelial Sodium channel) is an ion channel present in the kidney regulated by aldosterone. Its role consists in sodium reabsorption; thus, an overexpression results in an increase in fluid retention; vice versa, an under-expression results in less water retention, less body fluids, and blood volume with a consequent decrease of BP levels in experimental models [75,76]. The antihypertensive effect has also been shown in several clinical trials. In fact, the administration of 730 mg/day of quercetin for four weeks reduced systolic and diastolic BP in stage 1 hypertensive patients and had no effect in prehypertension patients. Moreover, the administration of 150 mg/day of quercetin for five weeks in patients with metabolic syndrome reduced systolic BP. The same BP-lowering effect was reported in type 2 diabetic women receiving 500 mg/day for 10 weeks of quercetin [77,78,79]. On the other hand, healthy subjects administered with 1 g of quercetin/day for 28 days showed increased quercetin plasma levels with no underlined cardiovascular benefit [80]. A meta-analysis revealed a statistically significant effect of quercetin on BP at doses higher than 500 mg/day [81]. Finally, an interesting study was conducted in 84 patients affected by gout and essential hypertension. The administration of quercetin to these patients, in association with standard therapy, induced echocardiographic changes. After a 12-month follow-up, these patients showed an improvement of cardiac diastolic function and of LV mass index associated with a better control of target levels of uric acid, of blood pressure control, and ultimately of renal function [82].

### 2.4. Curcumin

Curcumin is a yellow/orange vegetal pigment that has been associated with several beneficial cardiovascular effects [83]. Specifically, it has anti-oxidative, anti-inflammatory, and anti-proliferative effects in association with calcium homeostatic properties. These functions are associated both with a reduction of BP levels and prevention of vascular smooth cell proliferation [84]. In particular, in rat models, curcumin was able to prevent hypertension by lowering the expression of AT1R in arteries and by acetylation of GATA 4 [85,86]. Moreover, curcumin exerted a protective effect in nephrectomy-induced hypertension by enhancing the expression of Nrf2 [87]. The supplementation of 2000 mg/day of curcumin for 12 weeks in healthy middle-aged and older adults showed a cardioprotective effect mediated by an increase of NO bioavailability and by a reduction of oxidative stress [88]. Although no clinical trials are currently available to test the direct effect of curcumin in hypertensive patients, the latter study [88] highlighted a great potential of curcumin administration in this category of patients.

### 2.5. Berberine

Berberine is a vegetable alkaloid with many intrinsic properties. Some of its antihypertensive effects include both endothelium and vascular smooth muscle vasodilatation. The latter seems to be dose-dependent, and it results from augmented NO release (by eNOS increased expression), reduction of ACE effect, balancing of K^+^ channels activation, and intracellular Ca^++^ channels release [89,90,91,92]. A long-term protective action toward hypertension can also be detected, as berberine inhibits PDGF-induced vascular smooth cell growth and activation, reducing overall proliferation, thus antagonizing structural and functional vascular alterations [93,94].

Berberine exerts a beneficial effect on endothelial function as highlighted in a double-blind clinical trial carried in 23 patients, 12 of which received berberine for 1 month. In this study, berberine induced a reduced oxidative stress in the vascular endothelium by increasing the circulating CD31+/CD42- microparticles [95]. Consistently, 30 days of 400 mg berberine administration in healthy volunteers showed an upregulation of circulating EPCs and a relative increase of NO production [96]. An interesting randomized, double-blind, placebo-controlled study in 50 hypercholesterolemic patients showed an important decrease in total cholesterol levels, a relative reduction in LDL-c and triglyceride levels, an increased insulin sensitivity, and an improvement in endothelial-dependent flow-mediated dilatation (FMD) [97].

Although few studies are available to document the effectiveness of berberine toward an improved overall cardiovascular risk [98], no clinical trials are currently available to test the effect of berberine specifically on hypertension.

### 2.6. Brassica

Brassica is a novel compound in the landscape of antihypertensive nutraceuticals. It mainly acts as an antioxidant. It also induces ACE and renin inhibition, as reported in vitro [99,100]. Interestingly, these effects were most effective toward the hypertensive renal and cerebrovascular damage in an animal model. In fact, brassica administration for 1 month enhanced the AMPK/SIRT1/PGC1α/PPARα/UCP2 axis and induced strong renal and cerebral protective effect in the stroke-prone spontaneously hypertensive rat model (SHRSP) [101]. However, no reduction of BP was observed in this model upon brassica administration, suggesting that renal and brain protection were independent of systemic BP regulation, being likely the consequence of molecular mechanisms at the vascular level.

A clinical trial has analyzed the effects of Brassica intake on BP levels modulation. Interestingly, it was demonstrated that subclinical hypertensive patients showed a BP effect of brassica when they were a carrier of a GST genetic polymorphism, the GSTT1. Patients carrying the GSTT1 showed a better response [99]. However, in a randomized clinical trial with 40 hypertensive patients, the administration of 10 g of dried broccoli sprouts for 4 weeks did not significantly improve BP [102]. Although more clinical studies are needed to better highlight the true effectiveness of brassica in larger and more heterogeneous human samples, the animal model of salt-loaded SHRSP has so far provided encouraging evidence about a prompt and long-term effectiveness of brassica in the prevention of renal and cerebral hypertensive damage [101].

### 2.7. Spirulina Platensis

Listed as a “superfood of the future” [103], *Arthrospira* Platensis, better known as Spirulina, is a blue-green cyanobacteria that is currently a top-sold nutraceutical. This microalga contains numerous bioactive compounds [104]. In particular, peptides encrypted in the parent proteins become active after hydrolysis, which is operated by endogenous or other enzymes. The resulting peptides have shown antihypertensive activity. Among them, several tripeptides showed an inhibition of ACE in spontaneously hypertensive rats (SHR) in a concentration ranging between 11.4 and 315.3 µM. In particular, the tripeptide Ile-Gln-Pro showed a very potent activity similar to captopril with a decrease of systolic BP of 167.3 ± 2.5 mmHg [105]. This effect was demonstrated after both acute and chronic treatment by feeding SHR rats with enriched food. In this regard, the mRNA expression level and protein/peptide concentration of the main components of the renin angiotensin system were significantly altered by Spirulina treatment in the myocardium: ACE, angiotensin II, and angiotensin II type 1 receptor were downregulated, whereas angiotensin II type 2 receptor, angiotensin converting enzyme 2, angiotensin (1–7), and Mas receptor were upregulated. [106]. In addition to these short tripeptides, a heptameric peptide, namely Thr-Met-Glu-Pro-Gly-Lys-Pro, inhibited the Ang II-induced production of NO and of reactive oxygen species in human endothelial cells, and it downregulated the expression of inducible NO synthase (iNOS) and of endothelin-1 (ET-1) [107]. Recently, our research group has demonstrated that the gastrointestinal digestion (GID) of *Arthrospira* Platensis is able to evoke a dose-dependent vasorelaxation in murine models. In addition, a single decameric peptide identified in the GID of *Arthrospira* Platensis is able to exert important cardiovascular properties both in normotensive and hypertensive experimental models by promoting endothelial vasorelaxation and a reduction of BP levels through PI3K/Akt/eNOS signaling. These data could lay the basis for the future development of non-pharmacological products aimed at containing vascular dysfunction in different cardiovascular diseases [108]. Interestingly, a double-blind, placebo-controlled, randomized trial was carried in overweight hypertensive Caucasian patients to highlight the effect of spirulina consumption on BP control and endothelial function. In this trial, 40 patients received 2 g of either spirulina or placebo for 3 months. Subjects assuming spirulina showed a significant reduction of both systolic BP and stiffness index, documenting the important vascular effect of spirulina [109]. A similar study was conducted by administering 4.5 g of either spirulina or placebo in 16 patients with systemic hypertension. The spirulina group showed a significant decrease of systolic BP, along with a decrease of sVCAM-1, sE-selectin, and endothelin-1 levels, and an increase of glutathione peroxidase activity and oxidized glutathione levels, thus highlighting the spirulina antihypertensive and antioxidant effects [110]. A large-scale meta-analysis reported that spirulina reduced diastolic BP by a mean of 7.7 mmHg and reduced plasma lipid levels [111]. A clinical trial associated the spirulina vascular effect and blood pressure reduction in humans to both increased tone-related factors (in particular NO and vasodilating cyclooxygenase-dependent products of arachidonic acid) and to reduced level of vasocostricting eicosanoids [112]. Finally, a preliminary report on spirulina in a sample of the Mexican population highlighted a significant reduction of systolic and diastolic BP accompanied by a significant reduction of serum lipid levels, thus contributing to a major decrease of the overall cardiovascular risk [113].

**Figure 1:**
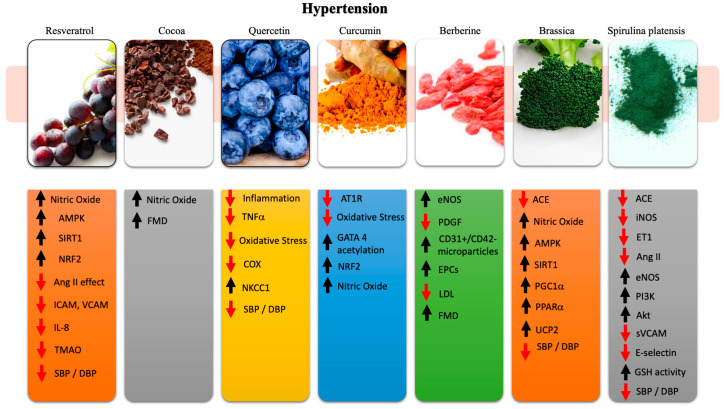
Summary of the main molecular and physiological effects exerted by resveratrol, cocoa, quercetin, curcumin, brassica, berberine, and Spirulina platensis in hypertension. Red arrow (↓) indicates downregulation; black arrow (↑) indicates upregulation.

The main effects that the nutraceuticals discussed play in hypertension are reported in Figure 1.

## 3. Nutraceuticals in Atherosclerosis

Atherosclerosis is a multifactorial degenerative disease that affects medium to large caliber arteries, inflaming and stiffening them due to the deposition of oxidized lipid and white blood cells in their wall, which typically occurs in adulthood or advanced age [114]. These deposits (said atheroma’s or atherosclerotic plaques) are located in the most inner layer of the arteries, the one that is in direct contact with the blood [115]. Their behavior is dynamic; that is, the lesions evolve with time: they begin in childhood as lipid streaks (reversible) and over the course of a few decades tend to become atherosclerotic plaques, especially in people who are predisposed and lacking attention to risk factors prevention [116].

Clinically, atherosclerosis may be asymptomatic or may manifest itself, usually between age 40 and 60, with acute or chronic ischemic phenomena that mainly affect the heart, brain, lower limbs, and intestines [117,118,119]. These ischemic phenomena are the consequence of the total or partial obstruction of the arteries affected by the atherosclerotic process [120,121]. In the process of arterial obstruction, platelets play a predominant role by adhering to damaged vessel areas, triggering the formation of occlusive thrombi or non-occlusive thrombi that contribute to the growth of plaques [122,123].

In Italy and many other countries in the world, atherosclerosis is one of the major health problems, which is mostly related to the lifestyle of industrialized societies [124]. In fact, atherosclerosis is either the cause or the contributory cause of serious diseases, such as angina pectoris, heart attack, and stroke [125].

Atherosclerosis is caused by the combination of several factors that can be grouped into two classes: systemic and local factors [126]. Systemic factors are represented by non-modifiable risk factors (age, gender, and genes) and modifiable factors (smoking, total hypercholesterolemia, low-density lipoproteins (LDL) and HDL cholesterol, hypertension, obesity, diabetes, hyperomocysteinemia, sedentary lifestyle, etc.) [126,127]. The simultaneous presence of multiple risk factors in the same individual has a negative cumulative effect [128].

The importance of local hemodynamic factors is demonstrated by the fact that although systemic factors affect the entire vascular system, atherosclerotic lesions are multifocal and typically develop in some areas (prone areas) [129]. This feature has been linked to the uneven distribution of mechanical stresses, whose intensity varies according to the composition and geometry of the vessel [130]. The lesions mainly affect the traits that are exposed to variations in hemodynamic forces: vessel curves, vessel bifurcations, and artery branches ostium [131]. With the aging process, the hemodynamic situation worsens, as the arteries become less elastic and the pulsatility of the flow accentuates (increasing the difference between systolic BP and diastolic BP) and extends toward the periphery [132,133].

Although atherosclerosis has been considered for a long time as a degenerative artery disease, over a century of studies has led to the conclusion that atherosclerotic lesions are the product of chronic inflammation of the intima leading to vascular dysfunction [134].

The initial events of the atherogenesis should be identified in the endothelial damage and in the accumulation and modification (aggregation, oxidation, and/or glycosylation) of low-density lipoproteins (LDL) at the interstices of the arteries, two events occurring prematurely and mutually reinforcing each other [123]. LDL accumulation is partly due to the increased permeability of the injured endothelium (functionally or anatomically), but mainly to their binding to the constituents of the extracellular matrix of the intima, which is a link that extends the residence time on site: adaptive thickening of the intima traps LDLs and increases the likelihood that they will be modified by free radicals produced by dysfunctional endothelium and leukocytes, as well as by hydrolyzed secretion enzymes produced by macrophages and smooth muscle cells (phospholipase A2 secretories, sphingomyelinase, and protease) [135,136]. The endothelial activation/dysfunction also involves the expression on the endothelial surface of adhesive molecules (selectin, VCAM-1, ICAM-1) and the production of chemotherapeutic signals inducing the adhesion and migration of monocytes and lymphocytes T in the intima, starting the inflammatory reaction [137]. Even smooth muscle cells located in the adaptively thickened intima exhibit adherent molecules on their surface (ICAM-1 and VCAM-1) and contribute to retaining macrophages in the lesions [138].

Knowledge of cardiovascular risk factors has made it possible to implement the primary prevention of atherosclerotic disease, which is aimed at limiting the exposure to them and, if the exposure is already established, to their treatment [139]. The measures to be taken for primary prevention include smoke control, excessive and overweight psycho-physical stress; proper nutrition; physical activity of at least 30 min per day 5 times a week; BP level within 140/90 mmHg; total serum cholesterol level within 190 mg/dl; blood glucose within 100 mg/dl at fasting and 140 mg /dl after a meal [140].

In this perspective, nutraceuticals may be of great value to help reducing risk factors in both a macroscopic way and in a pathophysiological way due to the importance of inflammatory and of oxidative pathways in atherogenesis [141].

### 3.1. Resveratrol

RES exerts several anti-atherosclerotic properties by acting on different pathways and mechanisms that are involved in this pathology [142]. RES downregulates the HMG-CoA reductase, which is the first enzyme involved in cholesterol biosynthesis, and it potentiates the effect of pravastatine [143,144]. Moreover, RES has been shown to decrease blood LDL cholesterol levels by reducing the expression of LDL receptors in hepatocytes [145,146]. Major factors involved in the atherosclerotic process are tackled by RES through its antioxidant properties, by inducing endogenous antioxidant pathways, by anti-inflammatory properties, and by the inhibition of smooth cell migration [147,148]. These effects are supported by the activation of SIRT-1, eNOS, Nrf2, antioxidant response elements, and by a decrease of TNF-α [149,150,151]. RES, through a reduction of the NF-κB pathway, can reduce the expression of intercellular adhesion molecule-1 (ICAM-1) and vascular cell adhesion molecule-1 (VCAM-1), which are associated with white blood rolling and intima inflammation [152,153]. On the other hand, the activation by RES of Akt and forkhead box O3a (FoxO3a) pathways in macrophages has been shown to decrease NADPH oxidase 1 expression and monocyte chemotactic protein-1 (MCP-1) production, ultimately reducing foam cell production [142,152,154]. Clinical trials show no direct activity of RES on lipid profile, as highlighted in a seven trial meta-analysis [45,155,156,157]. However, RES showed some beneficial effects in patients with type II diabetes mellitus and obesity. To be specific, the administration of 250–1000 mg RES/day lowered LDL cholesterol in type II diabetic individuals, whereas 150 mg and 500 mg RES/day lowered triglycerides in healthy obese men and in healthy smokers [158,159,160]. As stated before, RES works well when administered in association with other drugs, such as statins. In this combination, a 20% ox-LDL and 4.5% LDL cholesterol decrease was obtained with 350 mg RES/day in patients at high cardiovascular risk in primary prevention [161]. These data indicate that the pleiotropic properties of RES make this molecule an optimal candidate in association with many drugs that are routinely used.

### 3.2. Cocoa

Cocoa flavonoids, in addition to preventing cocoa butter rancidity, have an antioxidant effect also against free radicals produced by cellular metabolism. There are numerous polyphenolic compounds in cocoa seeds. Powdered cocoa is the food with the highest concentration of flavonoids: the concentration of cocoa and flavonoids is higher in the more bitter chocolate. In vitro studies have shown that flavonoids prevent the oxidation of LDL, blocking free radicals and chewing metal ions [162]. Flavan-3-ols-such as epicatechin bind to LDL and VLDL, protecting them from oxidation [163,164]. Monomeric and oligomeric flavan-3-ols have been identified as the main antioxidant compounds of cocoa [165]. Oligomeric procyanidins have shown hypocholesterolemic activity in rats fed with a high-fat diet by the inhibition of intestinal cholesterol uptake and increased fecal excretion [166]. Clinical trials involving the consumption of milk chocolate bars instead of a high-carbohydrate snack by young healthy subjects showed increased HDL cholesterol and decreased plasma triglyceride levels, but it did not affect LDL, despite an increased total fat in the diet [167]. A clinical trial showed a 12% reduction of serum total and LDL cholesterol levels with an intake of 100 g/day of polyphenol-rich chocolate for 2 weeks in hypertensive patients. Another trial using 75 g/day for 3 weeks in healthy subjects showed increased HDL cholesterol by up to 14% and an inhibition of lipid peroxidation [64,168,169]. In young adults, a decrease of total cholesterol level of 11% and of LDL cholesterol level of 15% was obtained after a short-term consumption of flavanol-rich chocolate [170]. Finally, an interesting study tested the effects of the administration of 100 g of dark chocolate (21.91 mg catechin, 65.97 mg epicatechin) vs. flavanol-free white chocolate for 15 days in 20 untreated hypertensive patients. As a result, an improvement of BP and vasodilatation levels as well as of LDL cholesterol level was observed [171]. The same result was obtained in hypercholesterolemic adults with a significant decrease of total serum cholesterol level of −6.5% and a −11.1% decrease of LDL cholesterol level [172]. Finally, a randomized clinical trial in 100 type 2 diabetic patients was carried with the use of 20 g of powder cacao/day for 42 days and it showed a reduction of total cholesterol, LDL cholesterol, and triglycerides [173,174]. Overall, the different outcomes suggest the need of larger well-controlled studies to understand the true benefits of cocoa in atherosclerosis.

### 3.3. Quercetin

Quercetin is found in high concentration in green leafy vegetables, nuts, flowers, barks, broccoli, olive oil, apples, onions, green tea, red grapes, dark cherries, blueberries, and cranberries [175]. In human trials, quercetin has been observed to possess anti-inflammatory properties by decreasing levels of CRP, thus acting as an important agent in the prevention of atherosclerosis [176]. Quercetin reduced blood LDL cholesterol levels and inhibited LDL oxidation by macrophages in overweight subjects with high cardiovascular risk [177,178]. Interestingly, these effects were associated with quercetin’s ability to induce both the maturation and apoptosis of human fat cells and to inhibit blood glucose uptake from fat cells in vitro. Mechanistically, the anti-inflammatory, anti-proliferative, and anti-atherosclerotic effects are mediated by the activation of SIRT1, which modulates the AMPK/NADPH oxidase/AKT/endothelial NO synthase signaling pathway [179] and by the suppression of ox-LDL-induced endothelial oxidative stress [180,181,182]. In Apo E knockout mice fed a high-fat diet (HFD), a model of atherosclerosis, quercetin suppressed ROS-induced ox-LDL production and inhibited p47phox levels [183].

To date, available clinical trials also show promising results. A randomized study in healthy patients with dyslipidemia, discovered by chance at blood tests, showed a decrease of total cholesterol, LDL, and triglycerides levels with a concomitant increase of HDL levels [184]. However, a meta-analysis of the effect of quercetin on lipid profile showed an effect only on triglycerides at doses above 50 mg/day [185]. However, in this meta-analysis, only five studies were eligible. Another meta-analysis provided a similar result by highlighting the effects of quercetin only on triglycerides level with a small to negligible effect on total cholesterol, LDL-c, and HDL-c levels, testing doses above 400 mg/day [185]. These evidences indicate the need for further studies to assess the true efficacy of quercetin.

### 3.4. Curcumin

Curcumin is the most effective anti-atherosclerotic nutraceutical known until now [186]. Curcumin decreases circulating LDL and increases HDL, with a 12% reduction of cholesterol level in healthy men. It shows other possible pharmacodynamic mechanisms [88,187]. In atherosclerosis, an overexpression of AR receptors is reported, and a curcumin derivative was shown to reduce AR expression [188]. The overproduction of lipopolysaccharides (LPS) in subjects with an atherogenic diets seems to be a cause of increased intestinal permeability, leading to glucose and atherosclerotic intolerance [189]. Oral supplementation with curcumin may be a potential therapeutic strategy to improve intestinal barrier function and prevent the development of metabolic diseases [190].

Curcumin has been shown to be effective in the reduction of total cholesterol and LDL levels also in 75 patients with acute coronary syndrome (ACS), through escalating doses of curcumin from 3 times 15 mg/day to 3 times 30 mg/day and finally to 3 times 60 mg/day [191,192]. An interesting meta-analysis of seven randomized controlled trials in patients with metabolic syndrome highlighted a significant improvement of fasting glucose, triglyceride, HDL-c, and diastolic BP [193]. Similar results were highlighted in randomized clinical trials in obese and overweight adolescent girls with 500 mg/day of quercetin [194]. However, in healthy subjects, contrasting results have been reported [195]. To date, there is a clinical trial that investigates the anti-inflammatory effect of curcumin on atherosclerosis with a 500 mg administration twice a day for 1 week (NCT02998918) [196]. Finally, a systematic review and meta-analysis of 20 randomized controlled trials with 1427 patients highlighted a significant decrease of triglycerides and elevation of HDL-c levels with no effect on total cholesterol and LDL-c levels [197]. These documented effects in association with the anti-inflammatory and antioxidant properties of curcumin make it a great candidate for atherosclerosis prevention and treatment [198].

### 3.5. Berberine

Controversial results have been reported regarding the efficacy of berberine in atherosclerosis [199,200]. Berberine lowers serum cholesterol levels in humans and hamsters through the induction of LDL receptors in hepatic cells, but at the same time, it induces foam cell formation in apoE^-/-^ mice, in RAW264.7 mouse cells, and in human primary macrophages, by inducing scavenger receptor A (SR-A) expression in macrophages with berberine-induced modified LDL uptake [201,202,203]. This double effect makes it difficult to evaluate the true overall positive effects of berberine. Thanks to the clinical research at our disposal, we can assess that berberine exerts an important protective effect on atherosclerosis in humans. A single-blind clinical investigation in 40 moderate dyslipidemic subjects with a berberine combination with other nutraceuticals for 4 weeks showed a significant reduction of total cholesterol by 16–20%, of LDL-c by 20–25%, of ApoB by 15–29%, and of triglycerides by 22–26% with an increase of LDL-c by 5.1–6.6% [204]. Another clinical trial was conducted with the combination of simvastatin plus berberine in 63 newly diagnosed hypercholesterolemic patients. A 2-month regimen with 500 g of berberine twice a day and 20 mg/day of simvastatin once a day demonstrated that the combination significantly lowered LDL-c level by an upregulation of LDL-r [205]. The berberine activity on LDL-r was also highlighted in a clinical trial in 32 hypercholesterolemic patients receiving berberine for 3 months [206]. Moreover, berberine was shown to reduce blood lipid level in hyperlipidemic patients with chronic hepatitis and liver cirrhosis [207,208]. More studies and trials are needed to assess the risk to benefit ratio and to further understand the involved pathways [201,203].

### 3.6. Brassica

Brassica is another agent that has been shown to function in many ways and to exert interesting effects. It was shown to reduce the overall serum cholesterol level without modifying the lipid profile and to reduce the LDL oxidation level in vivo in rats [209]. However, human trials have shown an interesting turn of events, linking the effects of brassica to glutathione S-transferase (GST) gene polymorphisms. In facts, an improvement of lipid profile was observed only in subjects carrying GSTT-1 [99]. Moreover, a reduction of platelet aggregation was also highlighted [99]. A clinical trial conducted with the administration of cold-pressed turnip rapeseed oil, high in brassica concentration, and butter highlighted a significant reduction of total cholesterol, of LDL-c, and of oxidized LDL levels in 37 subjects with metabolic syndrome. This improvement in serum lipid profile did not highlight significant changes in arterial elasticity [210]. This evidence shows the great potential of this agent. Its promising benefits need to be better assessed through additional trials.

### 3.7. Spirulina Platensis

Spirulina extracts have been used for the treatment of dyslipidemia. Some authors reported the effect of peptides, purified from gastric enzymatic hydrolysate of Spirulina, against early atherosclerotic responses induced by histamine in EA.hy926 endothelial cells. In particular, two tetrapeptides, Leu-Asp-Ala-Val-Gln-Arg and Met-Met-Leu-Asp-Phe, inhibited the production of adhesion molecules including P-selectin and E-selectin, and thus reduced the in vitro cell adhesion of monocytes onto endothelial cells [211]. Furthermore, Spirulina extracts were able to reduce total cholesterol serum levels, TG and LDL-C levels, while improving levels of HDL-C in both diet-induced hypercholesterolemic rabbit and mice [212,213], as well as in human trials [113]. However, in the latter studies, a clear identification of the main bioactive compounds is still lacking, although it is widely known that the numerous carotenoid molecules present in Spirulina ethanolic extracts [104] possess potent antioxidant and hypolipidemic activities. Although there are no direct clinical trials testing the effect of spirulina on the atherosclerotic process, there are encouraging trials on lipid profile changes in obese patients with cardiovascular risk factors. Among them, a randomized double-blind placebo-controlled study in obese patients with well-treated hypertension documented a significant improvement of BMI, waist circumference, and LDL-c concentration along with an improvement of insulin sensitivity [214]. Moreover, another clinical trial conducted in a similar cohort of patients showed even more promising results with a decrease of fasting plasma glucose, insulin concentration, reduction of total cholesterol, and of LDL-c with an increase of HDL-c [215]. Finally, another trial investigated the hypolipidemic effect of spirulina in association with physical activity in overweight and obese men. In the absence of physical activity, spirulina showed a consistent effect on lipid profile. When it was given in association with physical activity, the best outcome was achieved [216]. A similar study also showed that spirulina in association with physical activity can improve significantly cardiorespiratory fitness more than a single intervention alone [217]. A meta-analysis on the spirulina impact on plasma lipid concentration confirmed what was already known. Supplementation with spirulina can reduce total cholesterol, LDL-c, triglycerides, and increased HDL-c levels, heavily impacting on atherosclerosis [218]. The same consistent results were obtained in a randomized double-blind, placebo-controlled study in elderly Koreans and in a prospective study in a Cretan population [219,220]. Spirulina supplementation has powerful hypolipidemic and anti-inflammatory effects and should be advised in all patients with risk of atherosclerosis.

**Figure 2:**
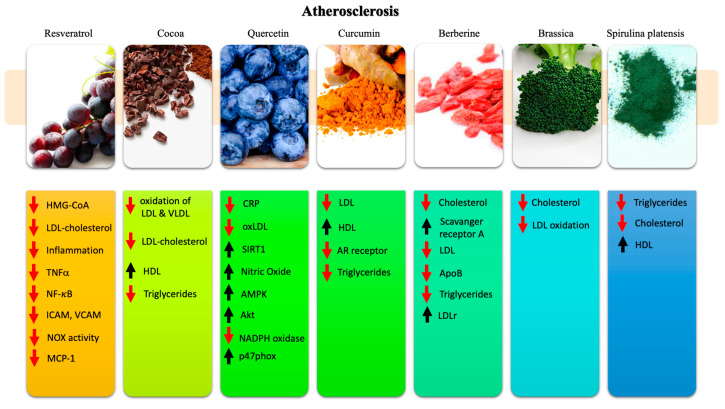
Summary of the main molecular and physiological effects exerted by resveratrol, cocoa, quercetin, curcumin, brassica, berberine, and Spirulina platensis in the atherosclerotic process. Red arrow (↓) indicates downregulation; black arrow (↑) indicates upregulation.

The main effects on atherosclerosis of the nutraceuticals reviewed here are reported in Figure 2.

## 4. Nutraceuticals in Heart Failure

Heart failure (HF) is a complex clinical syndrome defined as the heart’s inability to supply the blood in an adequate amount with respect to the actual requirement of the body [221]. Hemodynamically, heart failure is characterized by reduced myocardial contractility measured as the ejection fraction (EF), although this parameter does not indicate the cause of heart insufficiency [222].

According to statistics, with the aging population and the increase in the number of survivors of myocardial infarction, the incidence of heart failure continues to grow. In fact, incidence remains low in people between the ages of 40 and 50, rising to 10% in subjects over the age of 75 [223].

This condition can be caused by both organic and functional problems [221]. Among the most common causes are myocardial infarction, myocardial ischemia, hypertension, valvopathies, and cardiomyopathies [222]. Heart failure is the most important complication of any cardiac disease.

Cardiac failure is a clinical syndrome that manifests itself with a dysfunction of cardiac contractility (systolic dysfunction) and cardiac release (diastolic dysfunction) [224]. The subsequent and progressive activation of the neuro-endocrine system will coexist and overcome the insufficiency and congestion of the circulatory system. We could say that compensatory mechanisms aimed at maintaining an adequate tissue perfusion pressure and re-balancing blood supply to the various organs and apparatus may become the cause of further decompensation [221].

The treatment of HF consists in interrupting the feedforward cycle for which reduced ventricular function results in a reduced heart rate, resulting in neuronal activation (RAAS system) that causes peripheral vasoconstriction, fluid retention, and increased heart rate. This causes an increase in pre and post-load, myocardial hypertrophy, and adrenergic desensitization that ultimately worsens the initial condition [221]. In addition to the conventional pharmacological approaches, according to ACC/AHA classification with ACE-I, Sartans, and β-blockers in stage B, diuretics, or digoxin in stage C and LVAD, IABP, CRT, and ICD in stage D patients, stage A patients are recommended to follow a non-pharmacological approach including salt restriction, decreased fluid intake, and weight loss in obese patients and an increase of physical exercise. From this perspective, stage A patients are the best candidates for nutraceuticals, as they can partially interact both directly and indirectly with the mechanisms and the risk factors associated with HF [225,226].

### 4.1. Resveratrol

RES has been associated with several beneficial properties in reference to the pathophysiologic aspects of HF [41,227]. Apart from its anti-oxidant properties, achieved by enhanced SOD2 activation, increased antioxidant glutathione levels, and activation of eNOS, RES also inhibits, through AMPK activation and Akt inhibition, the protein synthesis in the cardiomyocytes. By doing so, it prevents left ventricular (LV) hypertrophy and improves heart rate and frequency through a better calcium cycling in association with the activation of the sarcoplasmic/endoplasmic reticulum Ca-ATPase 2a (SERCA2) expression via SIRT-1 [228,229,230]. RES has also reported to induce cardiac autophagy, which in turn contributes to reduce LV hypertrophy [231]. So far, these evaluations have been made in mice with evidence of a preventive effect slowing down the progression of heart failure [232]. Moreover, improvements in cardiac function and survival have been observed in mice with pre-existing HF conditions [230]. Of course, RES functions also on the structural and functional aspects of skeletal muscle and of vasculature, thus reducing HF symptoms, such as exercise intolerance and fatigue associated with muscles activity, and endothelial/vascular function. Furthermore, RES has shown cardioprotective effects in post-myocardial infarction (MI) HF experimental models by improving LV function, decreasing interstitial fibrosis, cardiac hypertrophy, and reducing oxidative stress markers [233]. Based on these evidences, RES promises to have a great value as a new therapeutic agent for HF treatment also in humans [234,235]. Unfortunately, there are very few publications on the direct effects of RES in HF. An interesting finding is the improvement of diastolic function and a low increase of systolic pump function in post-MI chronic coronary disease patients treated with 10 mg RES/day, accompanied by a decreased LDL cholesterol [236]. A similar indirect finding was obtained in a randomized, double-blinded, active controlled, parallel clinical trial by using 60-day oral supplementation of RES, where a 59.7% decrease of NT-proBNP was highlighted an important HF biomarker [237].

### 4.2. Cocoa

Cocoa, as previously discussed, is involved in several pathophysiological mechanisms associated with HF [238,239]. In healthy subjects, cocoa improves peripheral vasodilatation and the response to vasodilator factors [240]. In healthy subjects with one or more risk factors, cocoa has been reported to increase flow-mediated vasodilation and circulating NO, improve antioxidant status and platelets function, and decrease BP and LDL cholesterol levels. Cocoa supplementation also increases insulin sensitivity, induces coronary vasodilation, and improves coronary endothelial function and circulation (coronary velocity flow reserve) [66,67,241]. All these aspects, together with the effect of cocoa on BP modulation, as discussed above, are of great value for HF. Although more trials are needed to unify and better understand the efficiency level, cocoa con be already considered a new therapeutic candidate for HF patients and for all its associated CV risk factors [242].

To the best of our knowledge, there is no direct evidence of the protective effects of cocoa in HF. However, there is an interesting meta-analysis that investigates the correlation between chocolate consumption and risk of HF. This meta-analysis, including a total of five studies, showed that a moderate consumption of chocolate may be associated with a reduction of HF risk [243].

### 4.3. Quercetin

Quercetin is one of the most promising compounds for disease prevention. Although there are no studies regarding its direct effect on HF, quercetin remains a great compound with regard to the previously mentioned anti-inflammatory and antihypertensive properties [81,185,244]. The two combined effects are of great value for the prevention of CVDs that ultimately lead to HF. Although there are no direct studies associating HF and quercetin, an old epidemiological report showed a strong inverse association between mortality from coronary heart disease, stroke, and flavonoid intake, in particular quercetin [245]. In conclusion, a meta-analysis of these prospective cohort studies highlighted a reduction in coronary heart disease mortality in the top third compared to the bottom third of the dietary flavonoid intake curve [246].

### 4.4. Curcumin

Compared to quercetin, the beneficial direct and indirect effects of curcumin on HF have been well documented. By modulating the transcriptional p300/GATA4 pathway in cardiomyocytes, curcumin acts as a p300-specificc HAT inhibitor, preventing hypertrophy and reducing HF occurrence. A clinical trial in HF patients with hypertension and cardiac hypertrophy is currently ongoing, and the results are not yet available [247,248]. This new targeted therapeutic approach could be the first of its kind in HF. So far, the only available study with the use of curcumin in HF has been conducted in patients undergoing coronary artery bypass grafting (CABG). The administration of curcumin in 121 CABG patients showed a reduction in MI frequency. The latter was associated with the cardioprotective antioxidant and anti-inflammatory effects of curcumin, as highlighted by the reduction of post operatory levels of CRP, Pro-BNP, and BNP [249]. A double-blinded randomized, controlled trial in obese subjects with associated CVD risk factors showed not only the well-known effects of curcumin on lipid profile but also a reduction of homocysteine level, which is an important risk factor for HF [250]. Another study tried to evaluate the effect of curcumin in patients with unstable angina, in order to assess its role on HF prevention, as well as toward atrial and ventricular arrhythmias. However, curcumin doses of 80 mg/day for five days failed to exert cardioprotective effects, which was probably due to a short-term administration [251]. Further studies are needed to better evaluate the protective effects of curcumin in HF.

### 4.5. Berberine

Berberine indirectly influences HF outcome by lowering BP through adrenergic receptor blockade and by inhibiting acetylcholinesterase (AchE), which has a central sympatholytic effect. Moreover, it lowers blood glucose and low-density lipoprotein cholesterol and inhibits aldose reductase [252]. All these effects reduce cardiovascular risk factors prolonging the HF outcome [253,254]. The direct effect of berberine in HF is achieved through several mechanisms, such as the enhancement of coronary artery flow, the improvement of myocardial ischemia resulting from pituitrin, the amelioration of heart rate, the decrease in incidence of ischemic ventricular tachyarrhythmias, and by the modulation of inward and outward K^+^ rectifier currents, which prolong the action potential duration and then rapidly repolarize [255,256]. The first clinical trial that evaluated the effects of berberine in HF was conducted in 1988 in 12 patients affected by refractory congestive HF (CHF). An intravenous infusion of berberine at doses higher than 0.2 mg/kg per min showed a decrease of systemic and pulmonary vascular resistance, as highlighted by reduced right atrium and left ventricular end-diastolic pressures. Moreover, a reduction of chamber pressure was accompanied by increased cardiac index, stroke index, and LVEF at contrast angiography. These results were emphasized by echocardiographic indexes of overall systolic function [254]. A more recent trial on 156 patients with chronic CHF secondary to ether ischemic or idiopathic-dilated cardiomyopathy highlighted that the berberine treatment group (1.2–2 g/day) had an increased cardiac capacity with increased LVEF and increased exercise tolerance. The increased cardiac fitness was associated with reduced overall arrythmias and arrhythmic events. The overall mortality was decreased in the treatment group during a long-term follow-up [255].

Overall, these effects highlight a better heart efficiency and function. However, human trials are needed to evaluate the true efficiency and impact of this potential new therapeutic tool [254,257].

### 4.6. Brassica

No direct influence of Brassica has been documented in HF. Due to its previously mentioned properties, brassica can be of great value to prevent CVDs and ultimately HF.

### 4.7. Spirulina Platensis

Although there is no direct evidence on the effects of Spirulina in the protection from HF, the wide amount of antioxidant compounds included in its phytocomplex, such as carotenoids, peptides, and fatty acids [104], suggests that Spirulina may act in an indirect way. Its protective effect has been demonstrated in a model of doxorubicin-induced cardiotoxicity in mice. Doxorubicin, a well-known chemotherapeutic drug, possesses a severe cardiotoxic effect. In this regard, the pretreatment with Spirulina extracts showed lower mortality (26%), lower occurrence of ascites, lower levels of lipid peroxidation, and normalization of antioxidant enzymes as well as minimal structural damage to the heart [258]. There are no clinical trials testing the effects of spirulina in HF patients. The only trial worth mentioning was conducted in patients with ischemic heart disease. The supplementation of spirulina induced its well-known anti-inflammatory and hypolipidemic effects [259].

**Figure 3:**
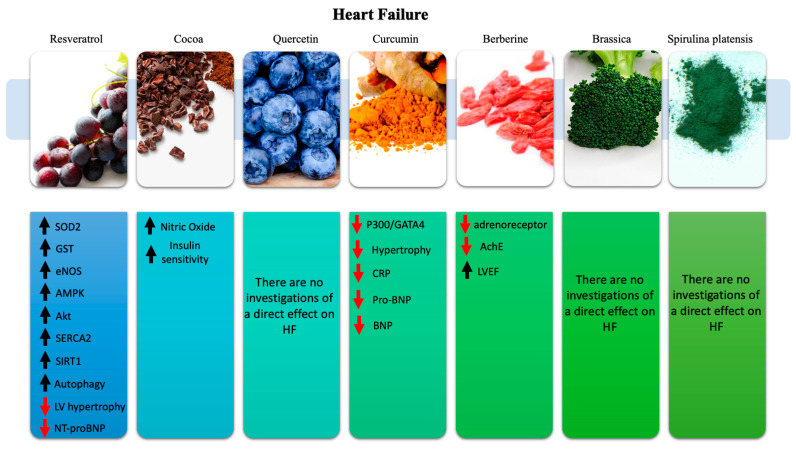
Summary of the main molecular and physiological effects exerted by resveratrol, cocoa, quercetin, curcumin, brassica, berberine, and Spirulina platensis in HF. Red arrow (↓) indicates downregulation; black arrow (↑) indicates upregulation.

The main effects exerted in HF by the nutraceuticals discussed in this review are reported in Figure 3.

## 5. Nutraceuticals in Diabetes

Diabetes mellitus is a chronic metabolic disease characterized by the presence of high blood glucose levels (hyperglycemia) due to a quantitative or qualitative deficit of insulin effect [260]. Diabetes affects 6% of the worldwide population, and 90% of these are affected by type II DM (associated with insulin resistance) [261]. The age prevalence of DM is different according to the type considered: type one DM has a mean age of about 15–29 years, whereas type II DM is prevalent at 60 years of age [262]. Accordingly, DM is also estimated to be the 8th cause of death worldwide and one of the most common comorbidities in the elderly [261]. DM is due to several causes, although ultimately, they all have the same mechanisms in common such as the alteration of glucose metabolism, insulin resistance, long/short-term insulin secretion, and an increased hepatic glucose output [263]. The decline of insulin activity and the hyperglycemia/hypoglycemia greatly affect the body in many ways. Acute medical complications, such as diabetic ketoacidosis and hyperosmolar hyperglycemic syndrome, may occur [264]. Of course, the long-term effects are of major interest, as they can be partially monitored, thus reducing death rates and unpleasant patient outcomes. Hyperglycemia causes macroangiopathy, which is a severe and early form of atherosclerosis, microangiopathy, and several vascular alterations of the small vessels [265,266,267]. Moreover, the diabetic alterations increase CVD and infection risk, jeopardizing the patient general state and long-term quality of life [268].

Treating and controlling DM is the number one priority for patient life expectancy and quality outcome. Guidelines in this sense greatly encourage the research of non-pharmaceutical molecules for borderline DM, as their effect in combination with a correct life style (diet and exercise) can be of great value for prevention [269,270].

### 5.1. Resveratrol

Compelling evidence demonstrated the anti-diabetic role of RES, both in animal models and in diabetic patients [271]. Glucose homeostasis is enhanced by RES by multiple mechanisms [272]. RES protects the pancreatic islands through anti-oxidant enzyme synthesis such as superoxide-dismutase, glutathione peroxidase, and glutathione-s-transferase, thus counteracting free radicals activity [273]. Interestingly, RES has also been proven to reduce B-cells apoptosis in animals with autoimmune insulitis of type I DM by reducing the expression of the IL-6 receptor and inhibiting the migration of inflammatory cells into the pancreas [274]. Moreover, RES is able to modulate hepatic and muscular glycemic homeostasis by reducing the activity of gluconeogenesis enzymes, increasing glycogen synthase, optimizing the fatty acid metabolism, and reducing the pro-inflammatory cytokines and protein synthesis [275,276,277]. In animals, RES has also been found to counteract adipogenesis and to reduce macrophage infiltration in fatty tissue, which is the main source of adiponectins. The latter are responsible for insulin resistance by the modulation of intracellular metabolic regulators such as SIRT1 and AMPK, which act on mitochondrial function and apoptosis [149,278,279,280,281,282,283]. All these beneficial effects of RES in animals have been translated also in primates and obese individuals. In this regard, a long-term treatment with RES was confirmed to improve insulin sensibility, reduce insulin resistance, and lower blood glucose levels [284,285,286]. The main issues regarding the therapeutic effect of RES in human trials relate to the correct dose, the time of treatment, and patient’s classes, as well as the low bioavailability and pharmacokinetics of RES. Given the concomitant presence of different pathologies in the metabolic syndrome, the employment of RES is very interesting. In this regard, a four-week supplementation of RES in a clinical trial of subjects with T2DM and coronary heart disease showed beneficial effects on glycemic control, HDL cholesterol levels, total/HDL cholesterol ratio, TAC and MDA levels, along with an upregulation of PPAR-γ and SIRT1 in the PBMCs of T2DM patients with coronary disease [287].

Another interesting placebo-controlled clinical trial, conducted with a 1 g RES dose for 45 days in type 2 DM patients, showed decreased fasting blood glucose, hemoglobin A1c, insulin, and insulin resistance values with a concomitant increase of HDL cholesterol, which are all specific anti-diabetic effects [288]. In DM patients, RES has been shown to be effective at lower doses as well. In fact, 4 weeks of RES at 10 mg/day reduced blood glucose levels and improved insulin sensitivity by decreasing oxidative stress, as shown by urinary ortho-tyrosine level reduction [289]. It is still uncertain whether low or high doses are more efficient. One meta-analysis of published trials highlighted a better efficiency of low doses, whereas another clinical trial showed no effect of RES at low doses [290]. Interestingly, a meta-analysis observed the impact of RES in diabetic patients but not in healthy subjects, highlighting a regulatory capacity of RES [291]. Of great clinical impact is the ability of RES to reduce foot ulcer size and increase the wound-healing rate, thanks to AMPK activation leading to improved vascularization of the wound bed and faster granular tissue deposition [292,293,294]. RES has shown to work wonderfully in combination with drugs. When combined with routine standard hypoglycemic medication, relatively low doses of RES (250 mg/day) showed an improvement of Hb1Ac, cholesterol level, and systolic BP [158,295]. More research is needed in order to fully assess the true value of RES utilization in clinical practice.

### 5.2. Cocoa

The impact of epicatechin, the main flavonoid of cocoa that is abundant in dark chocolate, has been demonstrated to affect insulin level and insulin resistance [296,297]. Although some mechanisms remain unclear, the beneficial effect on hepatic HepG2 cells by AMPK, SIRT-1/2, and PI3K/AKT pathways determining the activation of GLUT-2 transporters and enhanced insulin receptors phosphorylation ultimately enhances and strengthens the insulin signaling [298,299,300]. In human trials, 100 g/day for 15 days of flavonoid-rich chocolate in patients with essential hypertension led to a reduction of insulin resistance with an improvement of insulin sensitivity and beta-cell function [64,241,301]. A randomized placebo-controlled cross-over study conducted in 42 healthy, overweight, and obese patients with an administration of 500 mg per day of dark chocolate for 28 days induced a decrease of HOMA-IR and of fasting glucose [302]. Two interesting studies with a yearlong duration in 93 diabetic patients receiving high flavanol chocolate, 850 mg flavonoids day, showed a reduction of HOMA-IR and insulin levels as well as an increase of HDL level with an overall reduction of CVDs risk [303,304]. Finally, a randomized placebo-controlled, double-blind study of great practical clinical relevance was conducted in 60 diabetic patients receiving a high-polyphenol chocolate with 450 mg flavonoids for 56 days. This study highlighted a decrease in BP levels and, more interestingly, a decrease of both fasting glucose and HbA1c levels, which are important biomarkers of diabetic control routinely used in clinical practice for short-term management, fasting glucose, and long-term management of HbA1c [305]. Obviously, all cocoa components and their properties are beneficial to the overall health status, thus influencing indirectly insulin regulation in diabetic patient [301,306]. More studies are needed to fully understand the cocoa effects in humans as an anti-diabetic agent. It should be also interesting to evaluate whether cocoa is effective to reduce the cardiovascular complications of diabetes.

### 5.3. Quercetin

Quercetin has also been investigated as a possible anti-diabetic agent. In animal studies, it reduced levels of sorbitol, which is a sugar found in cells damaged by diabetes and affecting neuronal cells, kidney cells, eye cells, and in particular, endothelial cells [307]. Thus, the effectiveness of quercetin in animal studies appears quite interesting. The main antidiabetic mechanisms of quercetin include lipid peroxidation, increased SOD, GPX, and CAT enzyme activation, and inhibition of insulin-dependent PI3K activation [308,309]. Moreover, quercetin appears to stimulate AMPK-induced GLUT4 translocation and expression in skeletal muscle, thus reducing insulin resistance [310]. In combination with its antihypertensive and anti-oxidant properties, quercetin may be of great use in glucose tolerance control and postprandial blood glucose reduction [311,312]. As of today, there are no studies to assess the role of quercetin in insulin resistance in humans. Insulin resistance is one of the crucial agents in diabetes development. Conflicting results can be found in the two available epidemiological studies conducted with quercetin in type 2 DM. One study was performed in 10,054 women and men during a one-year lapsus and showed an association between higher quercetin intake and a reduction of type 2 DM. This result was accompanied by a total reduction in mortality from CVDs, lung disease, and some cancer types [313]. The other epidemiological study, which was conducted in 38,018 women aged above 45 years, showed no reduction in the development of type 2 DM [314]. The evaluation of quercetin effects on type II DM in humans needs more evidence in order to understand its full extension and applicability.

### 5.4. Curcumin

Curcumin is an effective nutraceutical agent against diabetes [315]. It indirectly positively affects diabetic-induced myocardial and vasculature damage [316]. The symptoms of diabetic cardiovascular myopathy can be attenuated by the anti-oxidant properties of curcumins, such as decreased activity of NAD(P)H, downregulation of JNK, and increased expression of cardiac metallothionein, ultimately increasing NO availability [317,318,319]. Moreover, diabetic cardiovascular myopathy symptoms can be slowed down by the anti-inflammatory properties of curcumins, which include a reduction of glucose-induced over-expression of TNF-α and IL-6 and suppression of JNK/NF-κB [320,321,322]. Curcumin is ultimately associated with a reduction of pro-inflammatory cytokines and level of oxidation, thus presenting as an anti-diabetic agent [323]. Clinical trials are scarce. One study performed in healthy subjects assuming 6 g of curcumin reported an increase in postprandial serum insulin levels but with no effect on plasma glucose levels. Another study showed a clinical improvement of visual function in patients with and without diabetic retinopathy [324,325]. A randomized, double-blind clinical trial carried in 53 type 2 diabetic patients with three times a day 1500 mg of curcumin for 10 weeks showed a significant change in mean weight, BMI, waist circumference, and fasting blood glucose level with no difference in HbA1c or HOMA-IR [326].

### 5.5. Berberine

Berberine, similar to curcumin, has been associated with antioxidant and anti-inflammatory properties [327,328]. Its therapeutic contribution to DM and insulin resistance can be explained by the effects on multiple signaling mechanisms such as AMPK, MAPK, Nrf2/HO pathway, and NF-κB pathway [329,330,331]. Of note, berberine has been proposed as a new possible drug for type II DM in humans [329,332]. It has a potent oral hypoglycemic property and a modest effect on lipid metabolism. Moreover, it is safe and of low cost, becoming largely distributable in low-economic social environments [93,333]. A study performed in 84 patients with type II DM initially treated with diet therapy and subsequently treated with either 500 mg 3 times a day of metformin or 500 mg 3 times a day of berberine or 500 mg 3 times a day of berberine plus previous anti-diabetic treatment for 3-months showed a hypoglycemic effect of berberine comparable to that of metformin, with a significant decrease of hemoglobin A1c, fasting blood glucose, and postprandial blood glucose levels. This antidiabetic effect of berberine was accompanied by a reduction of triglycerides and cholesterol levels, which is an effect that is not observed upon metformin administration. Moreover, berberine in association with other anti-diabetic therapy contributed significantly to achieve a control of hemoglobin A1c, fasting plasma insulin, insulin homeostasis, and cholesterol levels [334]. Another interesting study highlighted a specific effect of berberine which consists of an increase of insulin receptor expression in humans. This study, similar to the previous one, showed similar effects of berberine and metformin, with an additional effect of berberines on triglycerides level reduction [335,336]. Another study carried out in 116 type II DM patients, receiving 1 g berberine a day, highlighted similar effects as shown before such as a reduction of fasting and postprandial plasma glucose levels, reduction of HB1Ac, triglycerides, and cholesterol levels [327]. Although these studies are encouraging, a larger and more heterogeneous diabetic population trial is needed to assess the effectiveness and possible side effects of berberine [337].

### 5.6. Brassica

Brassica is of great interest for its multiple properties and is a good candidate as a new anti-diabetic drug [338]. In addition, due to its anti-oxidant and anti-inflammatory properties, brassica is effective in insulin resistance reduction, lipid metabolism, and profile correction [339,340]. Interestingly, brassica has a possible application for the prevention of diabetic neuropathy, which is mediated by Nrf2-dependent pathway, and for the prevention of diabetic vascular complications, which are mediated by p38 pathways, Nrf2 activation, and V-CAM1 suppression [338,339,341]. Of more clinical relevance, brassica shows a dose-dependent hypoglycemic property in animals with potential application to humans following clinical trials. A randomized double-blind clinical trial in 81 patients with type 2 DM, assuming oral administration of 10 g/day of broccoli sprouts powder for 4 weeks, showed a significant decrease of malondialdehyde (MDA) and of oxidized LDL-c levels and of oxidative stress index [338]. A similar study was conducted by using 10 g/day, 5 g/day broccoli sprouts powder or placebo for 4 weeks in 81 type II DM patients. This clinical trial showed that 10 g/day of broccoli sprouts powder is an effective dose to significantly reduce serum triglycerides, LDL, and ox-LDL levels and to increase significantly HDL-c level [342]. Finally, a randomized double-blind clinical trial on insulin resistance in type 2 diabetic patients documented, in agreement with the previous trials, that 10 g/day of broccoli sprouts powder for 4 weeks decreased significantly serum insulin concentration and HOMA-IR, yielding an important beneficial effect [343].

### 5.7. Spirulina Platensis

Spirulina possesses one of the highest contents of protein on dry weight [104], which is mostly represented by phycocyanin, a blue color protein. This protein showed promising hypoglycemic potential. In particular, C-Phycocyanin improves glucose production and phosphoenolpyruvate carboxykinase (PEPCK) and glucose-6-phosphatase (G6Pase) expression in HG-induced insulin resistant HepG2 cells. Furthermore, an increased glucose uptake, glycogen content and glycogen synthase (GS) activation in HG-induced insulin resistant HepG2 cells was observed. The underlying mechanism is probably based on the improvement of glucose homoeostasis via activating the IRS/PI3K/Akt and SIRT1/LKB1/AMPK signaling pathways [344]. Other authors showed a similar hypoglycemic effect in a diabetic rat model, in which Spirulina significantly lowered serum glucose, glycated hemoglobin (HbA1c), and malondialdehyde (MDA) levels while increasing (*p* > 0.05) serum insulin. These effects are probably due to the modulation of the MAPK pathway operated by antioxidant compounds such as carotenoids [345]. There are no clinical trials directly investigating the effect of spirulina supplementation in diabetic patients. Of interest, a reduction of fasting blood glucose level was reported in patients after spirulina supplementation, highlighting its potential use to increase insulin sensitivity [111]. Certainly, clinical trials are necessary to highlight the possible therapeutic outcomes.

**Figure 4:**
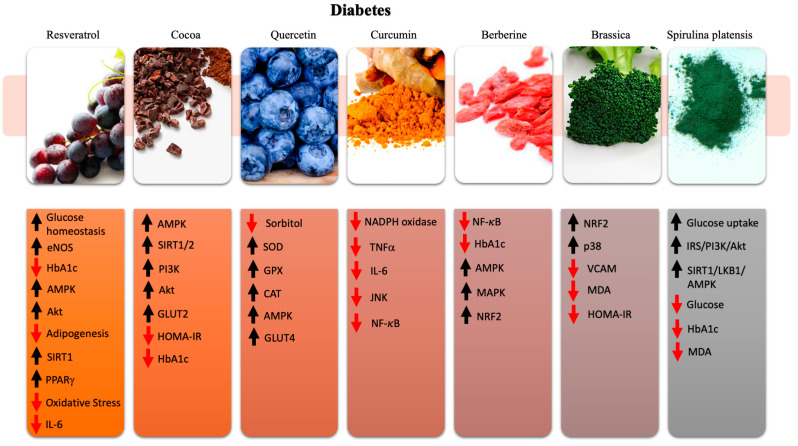
Summary of the main molecular and physiological effects exerted by resveratrol, cocoa, quercetin, curcumin, brassica, berberine, and Spirulina platensis in diabetes. Red arrow (↓) indicates downregulation; black arrow (↑) indicates upregulation.

The main effects exerted by the nutraceutical discussed here in diabetes are reported in Figure 4.

## 6. Discussion

**Figure 5:**
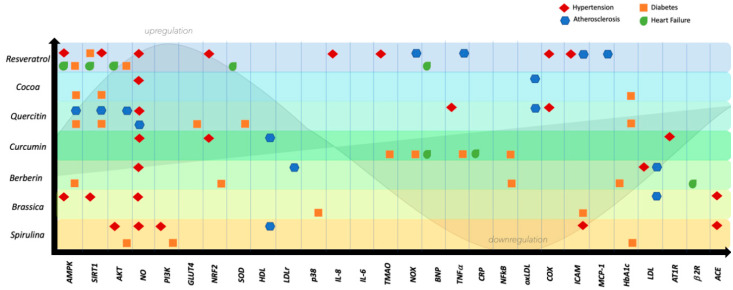
Schematic summary of the molecular effects of nutraceuticals in different CVDs.

In this review, we have focused our attention on the possible effects of some interesting nutraceuticals in different CVDs, highlighting their mechanisms of action and their potential in cardiovascular prevention. Although large and numerous clinical trials in humans are missing, with particular regard to some vascular diseases, the beneficial effects of these natural compounds have no shadow of doubt. The beneficial effects of resveratrol have been extensively described in several murine models, demonstrating its ability to reduce BP level, the progression of atherosclerosis, the regulation of glucose metabolism, and its effect on exercise tolerance and fatigue associated with HF in humans. Our review also summarizes the important effects of cocoa extracts on vascular function, highlighting its effects on endothelial vasorelaxation, on the reduction of cholesterol level, and on the activation of GLUT-2 transporters with enhanced insulin receptors phosphorylation in diabetes. On the other hand, quercetin, curcumin, and berberine exert their effects mainly through the activation of the antioxidant defense in all vascular diseases, and they help to protect from all major CVDs. Brassica vegetables, which include different kinds of cabbage, broccoli, cauliflower, Brussels sprouts, and kale, have recently received considerable attention due to their important effect on vascular protection, showing ACE inhibition and renin inhibition, along with the ability to modulate lipid profile, thus demonstrating significant protective effects on the vasculature. At last, but not least, Spirulina platensis appears to be the novel frontier of nutraceuticals, since it possesses several beneficial vascular properties, regulating NO release and vascular function and exerting an important effect in hypertensive murine models. Although the protective and therapeutic properties of resveratrol, cocoa, curcumin, and berberine are now well established through clinical trials, further studies are needed to characterize the efficacy and function of brassica and Spirulina platensis in humans. It should be also interesting to assess whether a combination of these nutraceuticals could be more efficacious than the single administration alone. In addition, taking into account the summary of the shared molecular targets (Figure 5), it should be interesting to evaluate in the future their possible interaction or potentiation. Moreover, it should be better to define which type of subject should be eligible for nutraceutical intervention. The studies reviewed here suggest that individuals at high risk to develop CVDs, rather than those with overt signs, may gain more benefits from nutraceutical interventions. For example, this class of subjects may include individuals with borderline values of blood pressure, as well as glucose or triglyceride levels. Another interesting future direction is to characterize how the physiological response to nutraceutics differs among individuals. To date, while all the listed nutraceuticals have several claimed properties to reduce or positively modulate the wide landscape of cardiovascular events, there is still a lack of knowledge of the molecular mechanism activated by this class of compounds. In this regard, the current and future direction of research is focused on the elucidation of the molecular pathway modulated by these molecules; in this context the employment of metabolomics, which can take the snapshot of a living system following a physiopathological perturbation, is one of the methods of choice. Metabolomics applied to nutraceuticals (Nutrametabolomics) will be the direction to understand in detail the complex link between the regular intake of phytochemicals and the healthy effect observed in several in vivo models. The future directions of nutraceuticals must take into consideration all the above-mentioned issues. In addition, another important question to be resolved with regard to nutraceuticals is their bioavailability and tissue accumulation after intake. Although for some of them, such as RSV, the available pharmacokinetic data show poor plasma bioavailability and fast metabolism after oral dose, while curcumin is instead detected in plasma in the form of glucuronide and sulfate conjugates in plasma, we have only few information regarding the absorption and the bioavailability of bioactive products following the intake of quercetin, brassica, cocoa, berberine and spirulina platensis.

In this regard, future studies are mandatory to elucidate nutraceutical pharmacokinetics, to highlight the correct posology of these products in order to selectively modulate the desired pathophysiological mechanisms. Furthermore, these data are necessary to validate several healthy claims of different nutraceutical products.

Moreover, we believe that future research must focus on clinical research. Understanding our target population and understanding therapeutic protocols for single, combined, and drug-associated nutraceuticals is fundamental. We hope that future research will answer some recurrent questions, such as: Who should we treat? What nutraceutical do we give? What posology? When do we start or stop treatment? These compelling future questions can be addressed with the use of translational medicine investigating the physiology behind nutraceutical mechanisms.

Certainly, based on their effects, nutraceuticals seem to be an important resource for the prevention and maintenance of vascular health before starting the therapeutic treatment or in association with it. Furthermore, the patient’s best predisposition to treatment with a natural substance could represent the best way to increase the preventive strategy and to reduce the incidence of CVDs.

Thus, it is reasonable to consider the use of nutraceuticals as complementary to the “traditional” lifestyle changes and “common” pharmacological therapies in order to improve the patient’s outcome.

## Abbreviations

CVD	cardiovascular diseases
DASH	dietary approaches to stop hypertension
ESC	European Society of Cardiology
ESH	European Society of Hypertension
LDL	low density lipoprotein
ox-LDL	oxidized-LDL
HDL	high-density lipoprotein
VLDL	very low-density lipoprotein
TG	triglycerides
BP	blood pressure
SP	systolic pressure
DP	diastolic pressure
RES	resveratrol
NO	nitric oxide
eNOS	endothelial NO synthetase
AMPK	adenosine monophosphate activated protein kinase
MAPK	mitogen-activated protein kinase
SIRT	sirtuins
NRF	nuclear erythroid factor
I-CAM	intracellular adhesion molecule
VCAM	vascular CAM
ANGIO	angiotensin
IL	interleukin
TMAO	trimethylamine-N-oxide
TNF	tumor necrosis factor
ENaC	epithelial sodium channel
LV	left ventricle
AT1R	angiotensin II type 1 receptor
ACE	angiotensin-converting enzyme
ACE-I	angiotensin-converting enzyme inhibitor
PDGF	platelet derived growth factor
CD	cluster of differentiation
EPC	endothelial progenitor cell
PMD	flow mediated dilatator
PPAR	peroxisome proliferator-activated receptor gamma
PPARGC1	PPARG coactivated 1 alpha
UCP	uncoupling protein
SHR	spontaneous hypertensive rat
SHRSP	SHR stroke-prone
GST	glutathione S-transferase
GID	gastrointestinal digestion
PI3K	phosphatidyl Inositol 3-kinsa
HMG-CoA	3-hydroxy-3-methyl-glutaryl-coenzyme A reductase
KFKB	nuclear factor kappa light-chain-enhancer of activated B cells
Fox03a	forkhead box 03a
MCP	monocyte chemotactic protein
NADPH	nicotinamide adenine dinucleotide phosphate oxidase
ApoE	apolipoprotein E
HFD	high-fat diet
LPS	lipopolysaccharides
ACS	acute coronary syndrome
SRA	scavenger receptor A
BMI	body mass index
HF	heart failure
CHF	congestive HF
EF	ejection fraction
LV	left ventricle
LVEF	left ventricle ejection fraction
LVAD	left ventricle assist device
RAAS	renin angiotensin aldosterone system
ACC	American College of Cardiology
AHA	American Heart Association
IABP	intra-aortic balloon pump
CRT	cardiac resynchronization therapy
ICD	implanted cardioverter defibrillator
SOD	superoxide dismutase
SERCA	sarco-endoplasmic reticulum Ca+-ATPase
MI	myocardial infarction
HAT	histone acetyltransferase
CABG	coronary artery bypass graft
CRO	C-reactive protein
BNP	brain natriuretic peptide
AchE	acetylcholine esterase
DM	diabetes mellitus
T2DM	type 2 DM
MDA	malondialdehyde
Hb1Ac	glycated hemoglobin
GLUT	glucose transporter
HOMA	homeostatic model assessment
HOMA-IR	HOMA insulin resistance
PEPCK	phosphoenolpyruvate carboxy kinase
GPX	glutathione peroxidase
JNK	c-Jun N-terminal kinases
IRS	insulin receptor substrate
LKB1	liver kinase B1
